# Variable clinical presentations of pulmonary hydatid cysts: a four-case series from a single center in United Arab Emirates, non-endemic region

**DOI:** 10.1093/jscr/rjag658

**Published:** 2026-07-29

**Authors:** Mahmoud Yehya, Mohamed F H Abdallah, Somar Ajeka, Fadi H Al-Masalmeh, Mhmad Othman J Albaghdadi

**Affiliations:** Department of Thoracic Surgery, SAQR Hospital, Al Juwais Al Kadi St, Ras Al-Khaimah, United Arab Emirates; Department of Radiology, SAQR Hospital, Al Juwais Al Kadi St, Ras Al-Khaimah, United Arab Emirates; Theodor Bilharz Research Institute, Mahad Al Abhas Al bahari St, Kornish Al Nile, Warraq Al Arab, Al Warak, Giza Governorate 3863341, Egypt; Department of General Surgery, SAQR Hospital, Al Juwais Al Kadi St, Ras Al-Khaimah, United Arab Emirates; Department of General Surgery, SAQR Hospital, Al Juwais Al Kadi St, Ras Al-Khaimah, United Arab Emirates; Kasr Alainy School of Medicine, Cairo University, Al Saraya St Old Cairo, Cairo Governorate 4240310, Egypt

**Keywords:** pulmonary hydatid cyst, echinococcosis, cystotomy, capitonnage, case series, United Arab Emirates

## Abstract

Pulmonary hydatid disease is an uncommon manifestation of Echinococcus granulosus infection in non-endemic regions and presents diagnostic challenges due to variable clinical and radiological features. We report a four-case series of pulmonary hydatid cysts from a tertiary center in the UAE. Presentations included hemoptysis, pneumonia-like illness with cavitary lesions mimicking malignancy, ruptured cysts with multi-organ involvement, and an incidentally detected large cyst. Diagnosis was established by imaging, supported by bronchoscopy, serology, and histopathology when indicated. Management was primarily surgical using lung parenchyma–preserving techniques, with staged procedures for extensive disease, and adjunctive albendazole therapy. All patients had favorable outcomes without early recurrence or mortality. This series highlights the diverse clinical presentations of pulmonary hydatid disease and underscores the need for clinical suspicion in non-endemic regions to ensure timely diagnosis and appropriate management.

## Introduction

Hydatid cyst is a long-known parasitic disease caused by the larval stage of *Echinococcus granulosis*, a type of tapeworm. Human cystic echinococcosis most commonly affects the liver; however, pulmonary involvement, the second most frequent site, can masquerade as any respiratory condition [1]. The chameleon nature of pulmonary hydatid disease is reflected in its wide spectrum of presentations, including life-threatening massive hemoptysis [2], eosinophilic pleural effusions mimicking allergic processes [3], community-acquired pneumonia [4], and space-occupying lesions resembling bronchogenic carcinoma [5].

Misdiagnosis carries significant consequences. In non-endemic regions such as the UAE, where cases mainly occur among male travelers and livestock-exposed populations, low clinical suspicion may lead to delayed diagnosis, inappropriate interventions, and preventable complications [6].

With early surgical management and appropriate postoperative anthelmintic therapy, pulmonary hydatid disease generally carries a favorable prognosis. Reported postoperative morbidity rates are up to 15%, with mortality not exceeding 2% in most series, particularly when lung-parenchyma–preserving techniques are applied in experienced centers [7].

## Case presentations

### Case 1

A 34-year-old male presented with intermittent hemoptysis for several months, associated with cough and mild dull right-sided chest pain. He had a history of hepatic hydatid cyst removal. Laboratory investigations were within normal limits.

Chest X-ray showed an ill-defined opacity in the right upper zone with a peripheral well-defined round lesion in the left lower zone, suggestive of pulmonary hydatid cysts given the history (Fig. 1).

**Figure 1:**
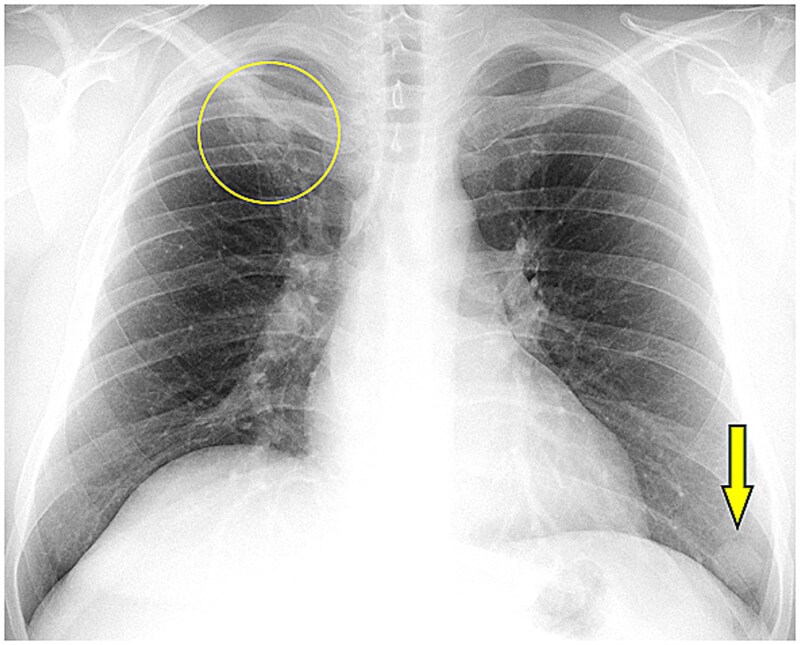
Chest X ray demonstrating a right upper lobe cavitary opacity (circle) and a well-defined rounded cystic opacity in the left lower lung zone (arrow down).

Chest computed tomography (CT) revealed a 3 cm right upper lobe cavity with a water-lily sign and a well-defined 2.5 cm cystic lesion in the left lower lobe, along with residual hepatic changes. Based on radiological findings and history, pulmonary hydatidosis was diagnosed.

He underwent lateral thoracotomy with cystotomy and capitonnage. The smaller intact left lower lobe cyst was managed medically.

The patient was discharged on postoperative Day 5 on albendazole (400 mg twice daily for 3 cycles of 4 weeks). A pre-discharge chest X-ray showed fully expanded lungs with residual findings (Fig. 2).

**Figure 2:**
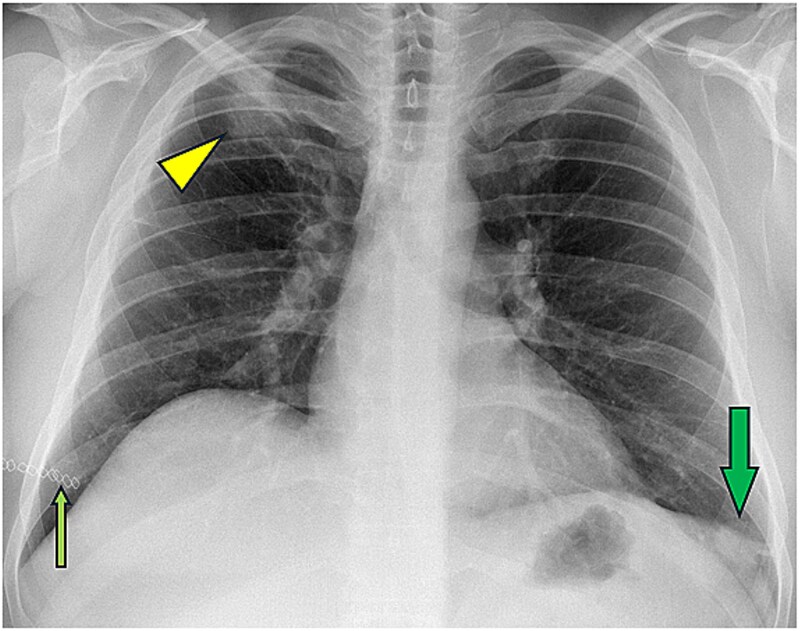
Postoperative chest X-ray showing right upper lobe opacity related to cavity closure (arrowhead) with residual left lower lobe hydatid cyst (arrow down). Skin staples also noted (small arrow up).

### Case 2

A 72-year-old female presented with right-sided chest pain, low-grade fever, chronic cough, and mild hemoptysis for 2–3 months.

Laboratory investigations were normal except for CRP of 45 mg/l (normal <5 mg/l).

Chest imaging revealed a right upper lobe cavitary lesion with thick irregular walls measuring 3 × 3 × 4 cm.

Given suspicion of malignancy, fiberoptic bronchoscopy was performed, revealing a whitish endobronchial membrane (evidence of germinal membrane of ruptured hydatid cyst).

She underwent right anterolateral thoracotomy, which confirmed a ruptured infected hydatid cyst. Cystotomy and capitonnage were performed. Histopathology confirmed an echinococcal cyst.

She was discharged on postoperative Day 9 on standard regimen of albendazole. Follow-up imaging showed recovery with residual linear consolidation.

### Case 3

A 28-year-old male presented with a sudden onset of cough producing clear salty sputum one week prior to admission, followed by fever and dyspnea.

Laboratory findings included leukocytosis (15.6 × 10^9^/l), neutrophilia (88%), CRP of 117 mg/l, and positive Echinococcus IgG (1:2560) normal range: Negative (<1:100).

Chest X-ray showed a cavitary lesion in the right lung and multiple mass-like densities bilaterally (Fig. 3).

**Figure 3:**
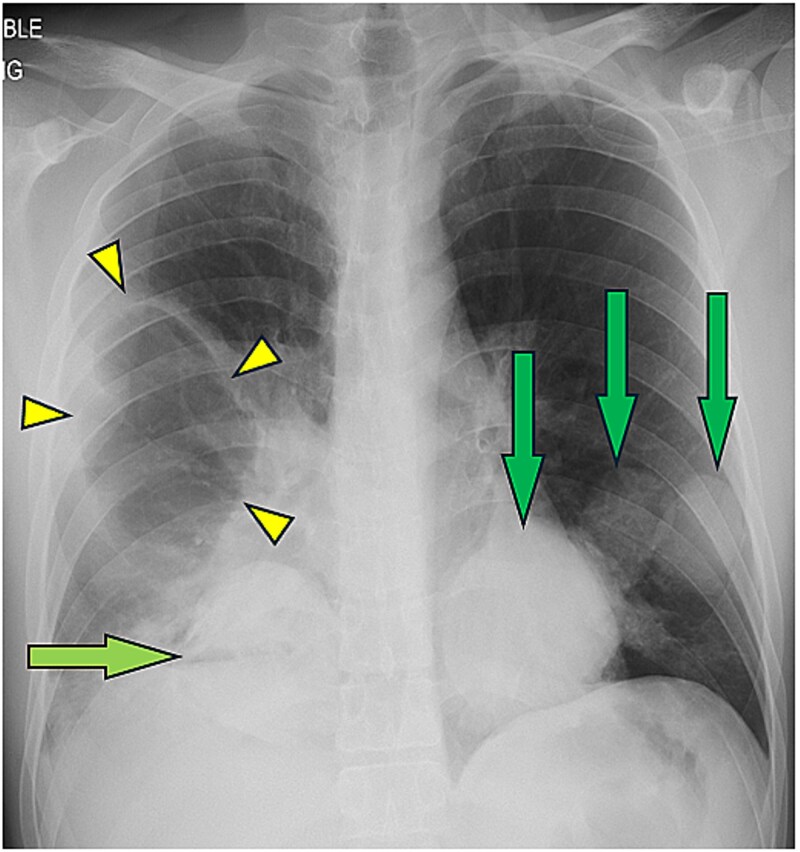
Chest X-ray demonstrating a cavitary lesion in the right upper/middle lung zone (arrowheads) with additional multiple rounded mass-like opacities involving the bilateral mid and lower lung zones (arrows), in keeping with multiple pulmonary hydatid cysts.

CT imaging demonstrated a ruptured hydatid cyst in the right upper lobe with surrounding consolidation, an intact cyst in the right lower lobe, multiple cysts in the left lung, and two large hepatic cysts (Fig. 4).

**Figure 4:**
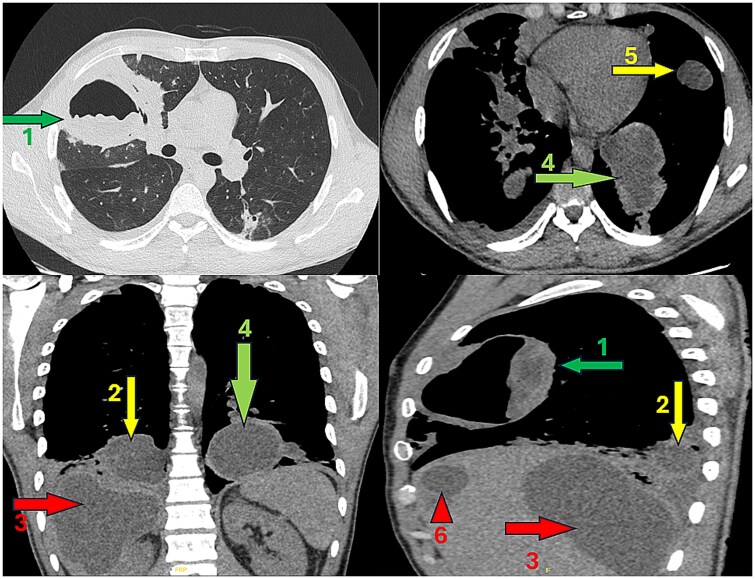
Chest CT images showing: [1] ruptured right upper lobe hydatid cyst with air–fluid level (water-lily sign), and surrounding consolidation, [2] intact right lower lobe pulmonary hydatid cyst, [3] giant hepatic hydatid cyst occupying the right hepatic lobe, [4] additional left lower lobe pulmonary hydatid cyst, [5] left upper lobe (lingula) pulmonary hydatid cyst, and [6] additional hepatic cystic involvement.

A sequential two phases surgical approach with 3-week interval was performed:

Phase 1: Right thoracotomy with trans-diaphragmatic removal of pulmonary and hepatic cysts.

Phase 2: Left thoracotomy for remaining pulmonary cysts.

Postoperative recovery was uneventful. Histopathology confirmed echinococcal cysts.

### Case 4

A 21-year-old male presented after a fall from height with polytrauma. He reported mild intermittent right-sided chest pain without other respiratory symptoms. Laboratory results were normal.

Chest X-ray showed a well-defined round opacity in the right lung (Fig. 5). CT revealed an incidental unilocular cystic lesion in the right middle lobe (Fig. 6).

**Figure 5:**
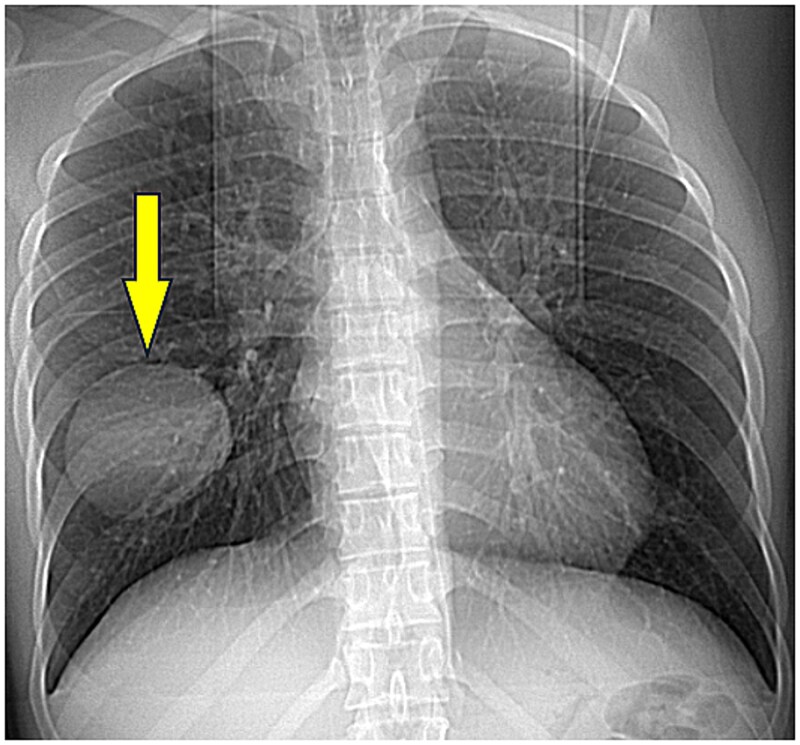
Portable chest X-ray demonstrating a well-defined rounded opacity in the right middle/lower lung zone (arrow down), suspicious for pulmonary cyst.

**Figure 6:**
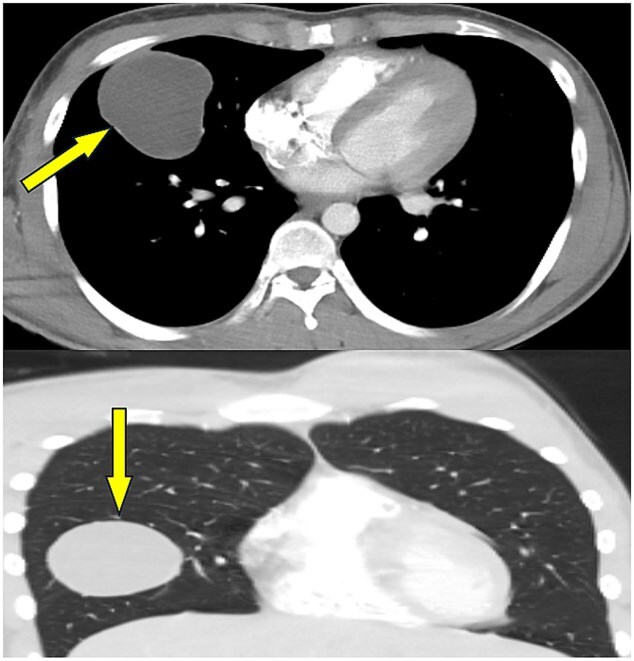
Trauma CT images demonstrating a well-defined unilocular cystic lesion within the right middle lobe (arrows), compatible with pulmonary hydatid cyst.

Suspected pulmonary hydatid cyst (>5 cm) was managed surgically. Starting with video assisted thoracoscopy (VATS) confirmed 7 × 7 × 6 cm intact hydatid cyst, followed by utility incision for lung-sparing enucleation and cavity closure (capitonnage) (Fig. 7).

**Figure 7:**
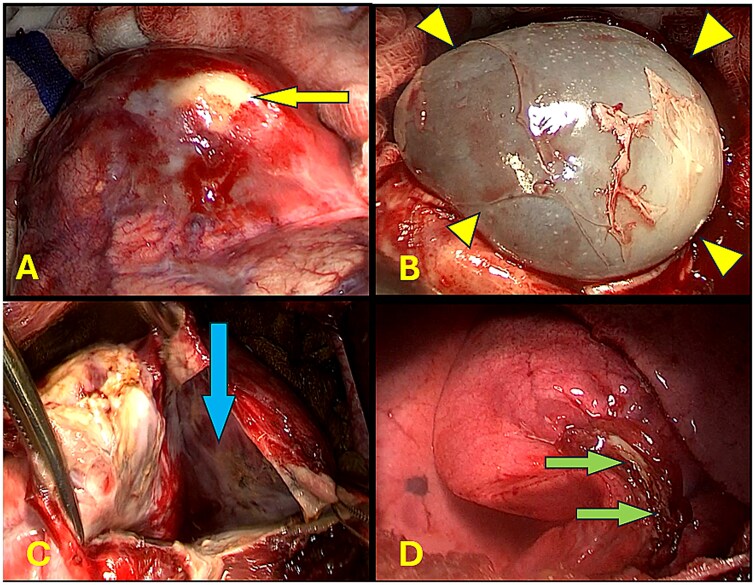
Intraoperative images: (A) Hydatid germinal membrane through middle lobe lung tissue (arrow left). (B) Intact hydatid cyst after peri-cystic dissection (arrowheads). (C) Intrapulmonary cavity after cystectomy (arrow down). (D) Line of tissue suture after capitonnage (arrow right).

Postoperative imaging showed good lung expansion with residual opacity at an obliterated cavity (Fig. 8). The patient was discharged after 6 days on albendazole therapy.

**Figure 8:**
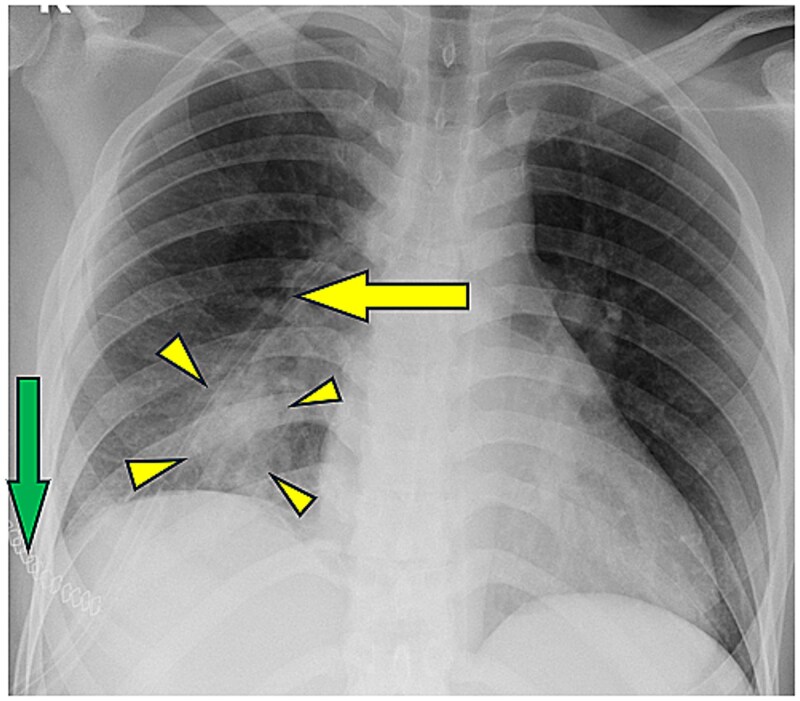
Postoperative chest X-ray showing expanded right lung with residual postoperative opacity at the site of the obliterated cyst cavity (arrowheads), line of chest drain (arrow left). And skin staples (arrow down).

## Discussion

Pulmonary hydatid cysts are uncommon in non-endemic regions such as the UAE and often present with nonspecific or misleading respiratory symptoms. Although no local prevalence data exist, studies from neighboring countries with similar demographics suggest that most cases occur among non-national males from endemic areas [6]. In our series, all patients were non-local nationals, 75% males consistent with this epidemiological pattern and highlighting how limited clinical familiarity can delay diagnosis.

### Clinical presentation and recognition

Although hydatid disease most commonly affects the liver (80.23%), pulmonary involvement remains significant (22.91%) [8] and should always be considered in patients presenting with chest pain (41.9%), cough (57.6%), hemoptysis (8.7%), and dyspnea (21.8%); some patients may remain asymptomatic (7.9%) [7, 8]. This emphasizes the broad clinical spectrum of disease.

The four cases in this series demonstrate this variability. Case 1 presented with chronic cough and hemoptysis, with prior hepatic hydatid disease serving as an important diagnostic clue. Given that hydatid disease frequently involves multiple organs and can recur at a rate of 4.6%–22.0% [9].

Case 2: is an elderly patient who presented with a pneumonia-like syndrome, chronic hemoptysis, and imaging suggestive of malignancy; diagnostic bronchoscopy showed the existence of an endobronchial germinal hydatid membrane and confirmed a chronic ruptured infected cyst.

Case 3: Shows the most acute presentation, with sudden production of clear salty sputum, pathognomonic of cyst rupture, followed by general symptoms.

In contrast, Case 4 represented an incidental diagnosis, with a large intact cyst (70 × 68 × 60 mm) identified during trauma imaging despite minimal symptoms.

These varied presentations highlight the need for maintaining clinical suspicion, particularly in patients with relevant exposure or travel history to endemic regions.

### Surgical management

Surgical resection remains the mainstay of treatment. The primary goals are complete removal of the parasite, prevention of intraoperative dissemination by covering the surgical field with sponges impregnated with hypertonic saline 30%, and preservation of lung parenchyma [7].

The surgical approach depends on the cyst size, location, and complexity. In most cases, cysts can be managed without lung resection using cystotomy and capitonnage [10].

Minimally invasive approaches (VATS) are suitable for selected, uncomplicated cysts, as in Case 4. Open thoracotomy was preferred in Cases 1 and 2 due to infection and suspected adhesions.

In Case 3, extensive bilateral pulmonary and hepatic involvement required a staged two-phase approach with a 3-week interval, allowing effective disease clearance while minimizing operative risk.

Cystotomy and capitonnage were successfully applied in three cases despite the existence of mild infected cavity in some cases, while enucleation and cavity closure were provided to one case with single intact cyst.

### Medical adjuvant therapy and outcomes

Albendazole may be used preoperatively in selected cases or postoperatively to reduce recurrence, particularly in cases of rupture, spillage risk, or multiple cysts. However, preoperative use may weaken the cyst wall and increase rupture risk [7].

All patients in this series received postoperative albendazole following the World Health Organization-recommended regimen: three 28-day cycles (10 mg/kg/day in divided doses) separated by 2-week intervals [11].

This regimen was effective, with no early recurrence observed, including complex and multi-organ disease [12].

### Clinical implications

Management of pulmonary echinococcosis requires both diagnostic vigilance and surgical expertise. Early recognition, whether through classic symptoms or incidental findings, allows timely intervention and prevention of complications.

This series underscores the importance of including hydatid disease in differential diagnosis even in non-endemic regions such as the UAE, where cases occur among travelers and livestock-exposed populations. Even in a low prevalence setting, pulmonary hydatid cysts can present diverse and atypical manifestations, often mimicking more common respiratory conditions. This case series highlights the importance of maintaining diagnostic vigilance in non-endemic regions to ensure timely recognition and appropriate management.

## Data Availability

All data supporting the findings of this report are included within the article.
